# Differences in muscle morphology assessed by ultrasound at various muscle regions and their impact on voluntary and involuntary force production

**DOI:** 10.1038/s41598-025-94158-z

**Published:** 2025-03-18

**Authors:** Marcel B. Lanza, Raziyeh Baghi, Nathan Frakes, Shabnam Lateef, Líbyna Thaynara Calandrelli Martins, Li-Qun Zhang, Vicki L. Gray

**Affiliations:** 1 Department of Physical Therapy and Rehabilitation Science, University of Maryland School of Medicine, Baltimore, MD USA; 2 Department of Health Science, Human Performance at Ithaca College, Ithaca, NY USA; 3 Department of Physical Therapy and Rehabilitation Science, University of Maryland, Baltimore, 100 Penn Street, Baltimore, MD 20201 USA

**Keywords:** Ultrasound imaging, Muscle thickness, Muscle quality, Muscle stiffness, Physiology, Musculoskeletal system, Skeletal muscle

## Abstract

The primary aim of this study was to investigate how measurements from different regions along the rectus femoris (RF) and vastus lateralis (VL) influence muscle morphology, including muscle thickness (MT), muscle stiffness, and muscle quality. An exploratory aim was to examine whether an association exists between voluntary and involuntary force and muscle morphology across the same regions. In one session, participants (*n* = 13) underwent ultrasound imaging (US), followed by knee extension maximal isometric voluntary contractions and evoked contractions. US recordings (at rest) and testing were conducted while participants were seated at 90º knee flexion (dominant leg) on an isokinetic dynamometer. Muscle morphology was recorded at proximal, medial, and distal regions of RF and VL. During maximum contractions, participants were instructed to exert maximal effort as fast and as forcefully as possible for 5 s, while evoked contractions were performed via femoral nerve stimulation. A one-way repeated measures ANOVA was used for the main aim, while Spearman bivariate correlations were used for the exploratory aim. The primary findings showed that the RF and VL muscles were significantly larger in the medial region (*P* ≤ 0.023), with no significant differences in muscle quality or stiffness within the same muscle. Additionally, a significant overall relationship was observed between muscle quality and the rate of force development in both muscles (*P* ≤ 0.037). In conclusion, muscle size varies across the length of the VL and RF muscles, with no changes in muscle quality or stiffness. Furthermore, muscle quality demonstrates a significant association with rate of force development.

## Introduction

The ability to produce maximal and rapid force is partially underpinned by muscle morphology, such as muscle size^1,2^, muscle quality^3^, and muscle stiffness^4,5^. All these muscle characteristics are often measured using ultrasound imaging (US). US is widely used for its non-invasive nature and real-time feedback capabilities for evaluating and studying soft tissue alterations, aiding in the identification of medical conditions, and understanding muscle morphology^6^. A major limitation of prior studies that used US to explore the influence of muscle morphology on force production is the measurement of a single muscle region^7–9^. This can be an issue, particularly given the possible change of muscle morphology (i.e., muscle size) across its length^10–12^. Muscle size varies along its length and may differ between muscles, but this has not been extensively studied. For instance, previous research laboratories showed an overall value of muscle size for the whole muscle group rather than specific muscles (e.g., quadriceps femoris)^12,13^. In addition to that, most past studies have primarily focused on muscle size. Thus, there is still a lack of clarity regarding how other aspects of muscle morphology (e.g., muscle stiffness and quality) and other relevant factors (e.g., subcutaneous tissue) change at varying muscle lengths. Additionally, the relationship between these measurements and force production remains unclear.

Muscle shape can vary along its length^14,15^; hence, it is reasonable to assume that muscle morphology may also alter across muscle length. Indeed, researchers have shown that some muscles have different sizes across their length^11,12,16^. Although the knee extensors are among the most studied muscle groups in sports and rehabilitation, there is limited evidence on how muscle morphology changes along the length of this muscle group based on muscle thickness measurements from US. This is important since US is a widely used technique due to its low cost and relatively easy manipulation^17^. Two studies used US to assess muscle size (muscle thickness, MT) of the quadriceps femoris and reported larger muscle size at more proximal sites compared to distal^12,13^. However, they did not report values from the other quadricep muscles. Another study demonstrated that the vastus lateralis (VL) and rectus femoris (RF) showed variations in muscle size along their length, as measured by cross-sectional area (CSA) via magnetic resonance imaging area^16^. The proximal and medial regions of the VL were larger than its distal location, whereas for the RF, the distal region was larger than the medial and proximal region^16^. Thus, the overall representation of muscle size from a muscle group may not accurately represent what happens at each muscle. There is a possibility that different measurement techniques, such as MT using ultrasound versus CSA using magnetic resonance imaging, may impact the outcome. Nonetheless, studies that measured muscle size using US at a single site of the quadriceps femoris and used it to infer the influence on force production may yield limited results^8,18–20^. This is due to possible muscle changes across different sites of the same muscle. Further understanding of how MT measurements across different regions of individual muscles of the quadriceps femoris influence force production is important.

Muscle quality and stiffness are two other important characteristics of muscle morphology, given the influence they have on force production^3–5^. US allows for the measurement of echo intensity, which has been used as an index of muscle quality^21^, while shear wave elastography enables the measurement of muscle stiffness^22^. Although both measurements have been widely used in different contexts, it is unclear how muscle quality and stiffness change across muscle length. One study has demonstrated that muscle stiffness changes across the RF muscle length, with stiffness decreasing from proximal to distal sites^23^. Other than that, the majority of studies have used a single measure of US from any of the quadriceps femoris muscles to determine muscle quality and stiffness^8,24–26^. For this reason, it is still unclear if muscle quality and stiffness change across the muscle length and its impact on the relationship with different manifestations of force. Given previous findings^23^ and the observation that muscle shape changes along its length^15^, it is reasonable to hypothesize that muscle composition may differ within the same muscle. Given previous findings and how muscle changes the shape within its length^15^, it is reasonable to hypothesize that muscle content could be different in different parts within the same muscle. This is a critical point to address because it is common for researchers from different laboratories to use different anatomical references when performing ultrasound measurements. For example, one research group performed RF US recordings at 50% of the horizontal line drawn halfway between the greater trochanter of the femur and the lateral knee joint line^25^, whereas another group used 50% of the distance between the proximal and distal myotendinous junctions of the leg^23^. Assuming that muscle stiffness and/or quality varies across different regions of a muscle, inconsistent anatomical landmarks may lead to measurement discrepancies between studies. Such variability complicates the integration of findings, limiting clinicians’ and researchers’ ability to fully interpret the phenomenon and apply it in evidence-based practice.

Therefore, this study has a twofold aim. First, to investigate how measurements from different regions along the muscle length of the RF and VL influence MT, muscle stiffness, and muscle quality measurements. Given the changes in muscle shape along its length and findings from previous research, it was hypothesized that these measurements would vary across the muscle length of the RF and VL. The second and exploratory, aim is to investigate whether an association exists between voluntary (maximal force and rate of force development) and involuntary force (twitch force) and muscle morphology (MT, stiffness, and quality) across different regions of the RF and VL.

## Results

### Differences in muscle morphology across muscle regions

**Fig. 1:**
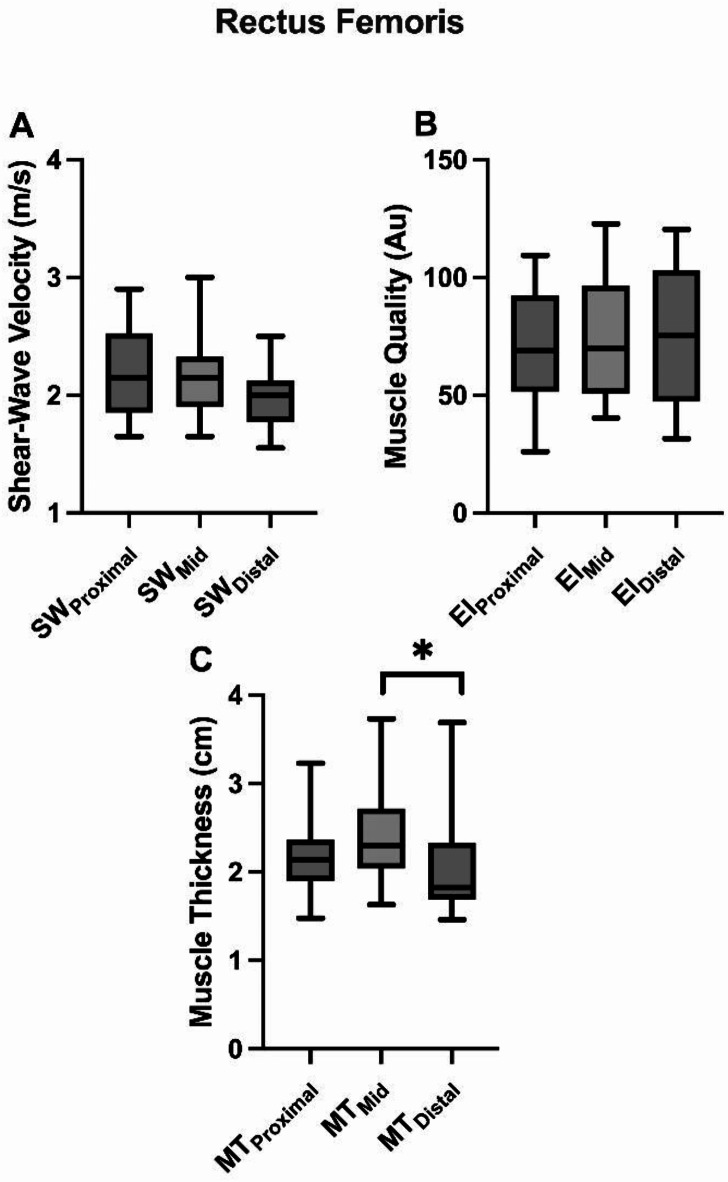
A comparison of (**A**) shear-wave velocity, (**B**) echo intensity (muscle quality), and (**C**) muscle thickness between different regions of the rectus femoris muscle. A symbol (*) indicates significant differences between means (*p* < 0.05).

**Fig. 2:**
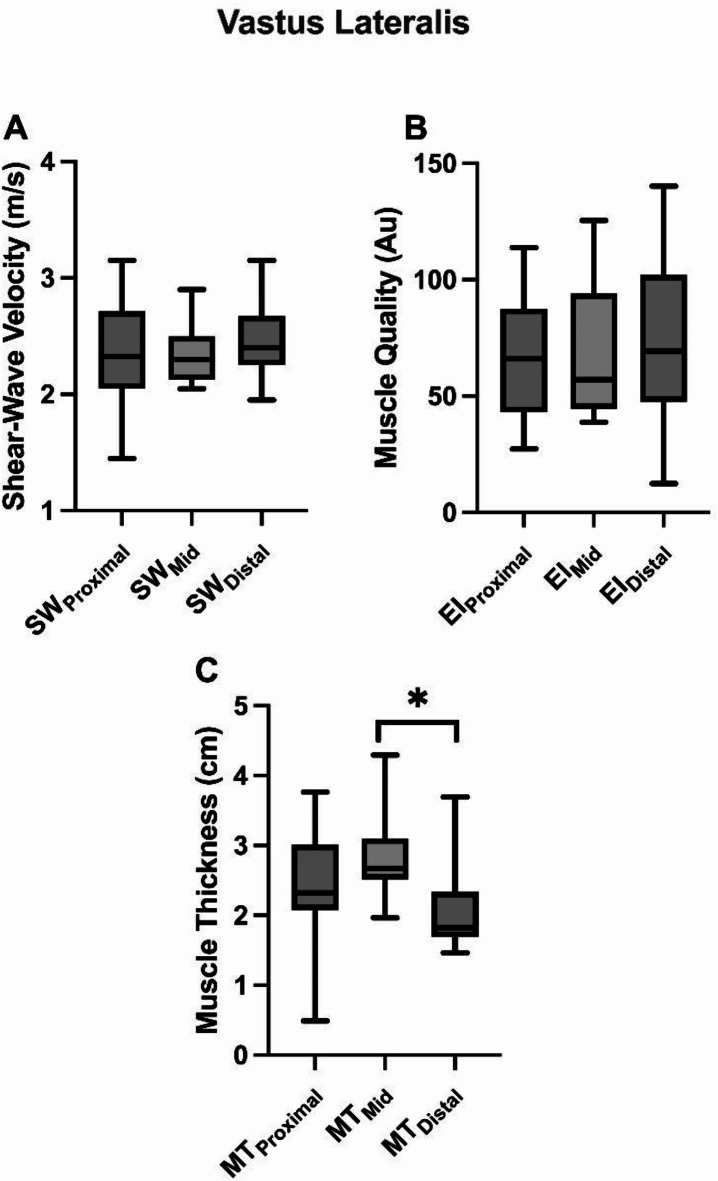
A comparison of (**A**) shear-wave velocity, (**B**) echo intensity (muscle quality), and (**C**) muscle thickness between different regions of the vastus lateralis muscle. A symbol (*) indicates significant differences between means (*p* < 0.05).

Rectus Femoris: The analysis revealed no significant differences across muscle length (proximal, medial, and distal) for muscle stiffness [F(2) = 3.199; *p* = 0.59; Fig. 1A] and quality [F(2) = 1.206; *p* = 0.317; Fig. 1B]. However, a significant difference was found between muscle size and the different locations [x² (2) = 8.769; *p* = 0.012; Fig. 1C], with the distal location being smaller than the medial location (Z=-2.272; *p* = 0.023, d = 0.52 “medium”). Vastus Lateralis: The results also revealed no statistically significant differences among the different muscle lengths when analyzing muscle stiffness [F(2) = 0.734; *p* = 0.492, Fig. 2A] and muscle quality [x² (2) = 2.923; *p* = 0.232, Fig. 2B]. Furthermore, a significant difference was observed between MT and muscle locations [x² (2) = 8.000; *p* = 0.018], specifically between the distal and medial positions (Z=-2.271; *p* = 0.023, d = 1.16 “large”), Fig. 2C.

### Association between force measures and muscle morphology

Rectus Femoris: No significant correlations were found between muscle stiffness at any muscle region with RFD, MF, and TF (*r* ≥ 0.072; *p* > 0.064), Table 1. However, muscle quality was significantly correlated with RFD at all muscle regions, with the lowest correlation found in the medial location (*r* = -0.687; *p* = 0.010), Table 1. Also, the mean value of muscle quality was also significantly correlated with RFD (*r* = -0.582; *p* = 0.037). MT (*r* ≥ 0.082; *p* > 0.078) and SF (*r* ≥ 0.104; *p* > 0.090) did not significantly correlate with any muscle force measures at any probe position.

**Table 1:** Bivariate correlations between rate of force development (RFD), maximum force (MF), and twitch force (TF) with muscle thickness, muscle stiffness, and quality from rectus femoris muscle.

	RFD		MF		TF	
	r	*p*	r	*p*	r	*p*
Rectus femoris						
Muscle stiffness proximal	0.113	0.713	0.072	0.816	0.143	0.640
Muscle stiffness medial	0.135	0.660	0.463	0.111	0.527	0.064
Muscle stiffness distal	0.416	0.157	0.427	0.146	0.201	0.510
Muscle stiffness all	0.184	0.547	0.303	0.315	0.369	0.215
Muscle quality proximal	**-0.637**	**0.019**	-0.313	0.297	-0.055	0.859
Muscle quality medial	**-0.687**	**0.010**	-0.478	0.098	-0.126	0.681
Muscle quality distal	**-0.588**	**0.035**	-0.335	0.263	0.209	0.494
Muscle quality all	**-0.582**	**0.037**	-0.418	0.158	0.050	0.986
Muscle thickness proximal	-0.093	0.762	0.082	0.789	-0.154	0.616
Muscle thickness medial	0.110	0.721	0.505	0.078	-0.033	0.915
Muscle thickness distal	0.165	0.590	0.148	0.629	-0.027	0.929
Muscle thickness all	0.220	0.471	0.258	0.394	0.022	0.943

Bold numbers indicate a significant correlation with *p* < 0.05. r = correlation coefficient, and p-values = level of significance.

Vastus Lateralis: No significant correlations were found between muscle stiffness at any muscle length with RFD, MF, and TF (*r* ≥ 0.086; *p* > 0.088), Table 2. Significant correlations were found between muscle quality and RFD at the medial and distal muscle locations (*r* = -0.791; *p* = 0.01; *r* = -0.637; *p* = 0.019), respectively. Similar to RF, the VL mean value of muscle quality was also significantly correlated with RFD (*r* = -0.659; *p* = 0.014). However, no correlation was found between muscle morphology and the other measurements of force (*r* = -0.352; *p* > 0.239). Additionally, both MT (*r* = 0.676; *p* = 0.011) at the medial muscle locations and SF (*r* = -0.571; *p* = 0.041) at the proximal muscle location showed significant correlations with MF. No other locations of MT and SF showed significant correlations, with MF (*r* ≥ 0.126, *p* > 0.071; *r* ≥ 0.176, *p* > 0.064; respectively), Table 2.

**Table 2:** Bivariate correlations between rate of force development (RFD), maximum force (MF), and twitch force (TF) with muscle thickness, muscle stiffness, and quality from Vastus lateralis muscle.

	RFD		MF		TF	
	r	*p*	r	*p*	r	*p*
Vastus lateralis						
Muscle stiffness proximal	0.182	0.571	-0.056	0.863	0.123	0.704
Muscle stiffness medial	0.086	0.780	-0.125	0.684	0.272	0.369
Muscle stiffness distal	-0.204	0.505	0.326	0.276	0.491	0.088
Muscle stiffness all	0.030	0.921	-0.155	0.613	0.199	0.514
Muscle quality proximal	-0.500	0.820	0.110	0.721	-0.132	0.668
Muscle quality medial	**-0.791**	**0.010**	-0.341	0.255	-0.033	0.915
Muscle quality distal	**-0.610**	**0.027**	-0.352	0.239	-0.104	0.734
Muscle quality all	**-0.659**	**0.014**	-0.214	0.482	-0.033	0.915
Muscle thickness proximal	-0.016	0.957	0.126	0.681	0.516	0.071
Muscle thickness medial	-0.143	0.642	**0.676**	**0.011**	0.346	0.247
Muscle thickness distal	0.165	0.590	0.148	0.629	-0.027	0.929
Muscle thickness all	0.093	0.762	0.187	0.541	0.412	0.162

Bold numbers indicate a significant correlation with *p* < 0.05. r = correlation coefficient, and p-values = level of significance.

## Discussion

We investigated whether measurements from different sites along the VL and RF muscles affect muscle morphology. Our hypothesis was partially confirmed, as MT of both the VL and RF varied along the muscle length; however, only the VL showed differences in muscle quality across locations. Muscle stiffness did not vary across locations for either muscle. Additionally, we aimed to explore the association between force production and muscle morphology. Our findings revealed an overall significant relationship between muscle quality and RFD for both muscles. Thus, muscle quality may influence how rapid force is produced, but it may not affect the maximal capacity to produce maximal or involuntary force.

The differences in muscle size across the various regions of the VL and RF are not surprising, as both muscles exhibit changes in shape from origin to insertion^15^. Previous research has reported changes in muscle size (CSA) for both the RF and VL across different regions of the muscle^16^. The authors showed the VL had a larger size at proximal and medial locations compared to the distal location, while the RF had a larger size at a distal location than its medial and proximal locations^16^. In contrast, we found higher muscle size at the medial region of the VL and RF compared to the distal region, while the proximal and distal regions were similar. Although the results are somewhat different, the pattern for the VL was very similar but it was the opposite for the RF when comparing studies. The difference in results could be due to the employment of different measurement techniques and equipment, which in this case was MT using ultrasound versus CSA using magnetic resonance imaging. Another possibility is the different populations between studies; we used a mixed sample of males and females, while the previous study^16^ only included females. Maybe there are differences in muscle morphology between males and females.

This is the first study to demonstrate that VL or RF muscle quality and muscle stiffness do not change across its length. This appears to be opposite to previous findings showing that RF muscle stiffness decreases from proximal to distal regions^23^. The discrepant findings might be due to the previous investigation only reporting a descriptive difference, with no actual statistical analysis performed to compare the values between regions of the muscle. Furthermore, because we identified changes in MT, these results suggest that the relative composition of connective tissue and fat may adjust accordingly within those muscles, yet not substantially affect muscle quality or stiffness. Consequently, for muscle quality and muscle stiffness, a recording of one site might be enough to represent what is happening at the whole muscle, which is not true for MT.

Muscle stiffness was not associated with voluntary (MF and RFD) or involuntary force (TF) in the present study. Opposite, Ando and Suzuki (2019) demonstrated that maximum torque and the ability to rapidly produce torque in the plantar flexors were associated with muscle stiffness of the medial gastrocnemius^4^. Additionally, another study examined the relationship between VL muscle stiffness and various lower-limb performance measures—specifically, vertical jumps, countermovement jumps, rebound jumps, and multi-joint leg extension power. The author reported mixed results, with certain performance parameters (e.g., rebound jump) showing a significant correlation with muscle stiffness, while others (e.g., countermovement jump) did not^27^. These contradictory findings may indicate that the relationship between strength and muscle stiffness is task-dependent, as we assessed an MVC while the other author examined jump tasks and dynamic contractions. Nonetheless, previous studies have shown that voluntary force (RFD and MF) is influenced by the level of activation of the target muscles, the size of the muscles, and the quality of the muscle^2,3,28,29^. This information, combined with the lack of association with involuntary force, suggests that RF and VL muscle stiffness is likely less influenced by the active contractile elements responsible for force production and is primarily determined by passive structural components, such as the extracellular matrix and connective tissues^30^. Future studies may explore this possibility.

The ability to rapidly produce force (RFD), was influenced by muscle quality in both muscles. Echo intensity, our measure of muscle quality, evaluates the gray-scale of ultrasound images, where skeletal muscle appears hypoechogenic (closer to black), while fibrous tissue and intramuscular fat are hyperechogenic (closer to white)^21^. Nonetheless, there is some controversy regarding the accuracy of echo intensity in measuring fibrous tissue and intramuscular fat. Considering this information, we demonstrated that muscles with higher quality—potentially containing less fibrous tissue and intramuscular fat—are capable of producing force more quickly. This finding aligns with a previous study, which also found no significant association between muscle size and rapid torque production but showed that muscles with lower fat content produce torque at a faster rate^31^. Hence, individuals aiming to produce force quickly might benefit from muscles with lower fat and connective tissue content.

In contrast to previous research, overall our data set did not show that larger muscles were able to produce higher MF or involuntary force. The only exception was the VL muscle size in the medial region, which showed a significant (moderate) association with MF. In accordance with our results, we found at least one study using ultrasound measurements that did not identify a relationship between muscle size and the strength of the knee extensors^32^. However, other studies have demonstrated that larger quadriceps femoris muscles are often capable of producing greater knee extension forces voluntarily^18,31,33,34^. These discrepant findings might be due to differences in methodology between our study and others. First, the associations in our study were analyzed for a single muscle, while the force measurements reflect the combined output of all four quadriceps femoris muscles. This may partially explain the overall lack of significant relationships in our results. Secondly, we used US images from a longitudinal view and measured RF and VL muscle thickness. There has been debate about the accuracy of ultrasound imaging in estimating muscle size^33,35^. The use of measurements from magnetic resonance imaging and/or computed tomography scans provides a more accurate assessment of muscle size. These imaging techniques allow for the measurement of the entire muscle at small intervals across its length (e.g., 4 mm slices), enabling detailed volumetric and cross-sectional area analyses. Although our study measured three different parts of each muscle, this approach was not enough to show a significant relationship between muscle size and strength. Future studies may use different techniques to assess quadriceps femoris muscle size, and verify its influence on the ability to produce voluntary and involuntary force.

Some limitations should be highlighted here. While we had sufficient statistical power to address the primary aim of our study, we did not have adequate power for the secondary aim. Thus, the findings related to the secondary, and exploratory, aim should be interpreted with caution. Future studies should replicate this portion of the experiment with a larger sample size to confirm or refute our findings. Furthermore, our rest interval between the MVC and the evoked contraction was at least 5 minutes. Although some evidence supports this duration^36^, we acknowledge it may not have been sufficient for certain participants. Nonetheless, we adopted this strategy to minimize participant drop-outs due to the discomfort caused by the electrical stimulus. While our participant pool is nearly evenly split between males and females—reducing the likelihood of significant gender bias—there may still be sex-related differences in our measurements. Unfortunately, our sample size was insufficient to determine this conclusively, and future research should further investigate potential gender-based disparities. In the present study, the test was performed at 90º of knee flexion. It is well-known that the ability of the knee extensors to produce force varies across joint angles. Hence, the findings we presented may differ if testing is conducted at different joint angles. Moreover, while we used the term “muscle quality” to describe echo intensity measurements, this technique reflects general tissue composition (e.g., fat, connective tissue, and muscle fibers) but does not differentiate between these components. This limitation reduces the specificity of muscle quality assessments. Therefore, to better understand the role of fat content and connective tissue in force production, researchers may employ imaging techniques such as magnetic resonance imaging and/or computed tomography scans, which are considered the ‘gold standard’ for these assessments^37,38^.

In conclusion, while the RF and VL muscles appear to be larger in the central region, there is no difference in muscle quality and stiffness within the different parts of the same muscles. Furthermore, although a significant overall relationship was found between muscle quality and the ability to produce force quickly in both muscles, neither muscle size nor stiffness influenced any of the force measurements performed.

## Methods

### Participants

Thirteen individuals (ages 18 to 35 years, Table 3) with no history of muscular disorders, lower extremity surgery, or serious injuries volunteered for this study. A total sample size of 5 participants was determined by using GPower (version 3.0.1, Germany) based on a test using an analysis of variance (ANOVA repeated-measures within factor), with an alpha level of 0.05, power of 0.80; effect size (ES) of 0.71 (based on the average effect size value among the three comparisons of muscle stiffness from previous study^23^), number of groups, 1; number of measurements, 3; correlation among measures of 0.5; and a nonsphericity correction of 1. All volunteers provided written informed consent before their participation, and approval was granted by the University’s Institutional Review Board. The experiment was conducted in accordance with Declaration of Helsinki.

**Table 3:** Characteristics of individuals are expressed as mean ± standard deviation.

*n*	13
Females	7
Age (years)	27.69 ± 2.49
Height (m)	1.68 ± 0.80
Body mass (Kg)	69.0 ± 21.1
BMI (Kg/m^2^)	24.1 ± 7.0
Maximum Force (N)	792 ± 392
Twitch Force (N)	115 ± 91.4
Rate of Force Development (N/s)	424 ± 279

### Overview

In one session, participants were subjected to ultrasound imaging, followed by knee extension maximal isometric voluntary contractions (MVIC), and evoked contractions. Testing occurred while participants were seated on an isokinetic dynamometer (Humac Norm, Stoughton, MA). All measurements were taken on the dominant leg. The leg dominance was defined by asking the following question: which foot would you kick a ball^39^? 

### Measures and procedures

Initially, participants signed the consent form, and then their height and weight were measured. 

#### Ultrasound measures

**Fig. 3:**
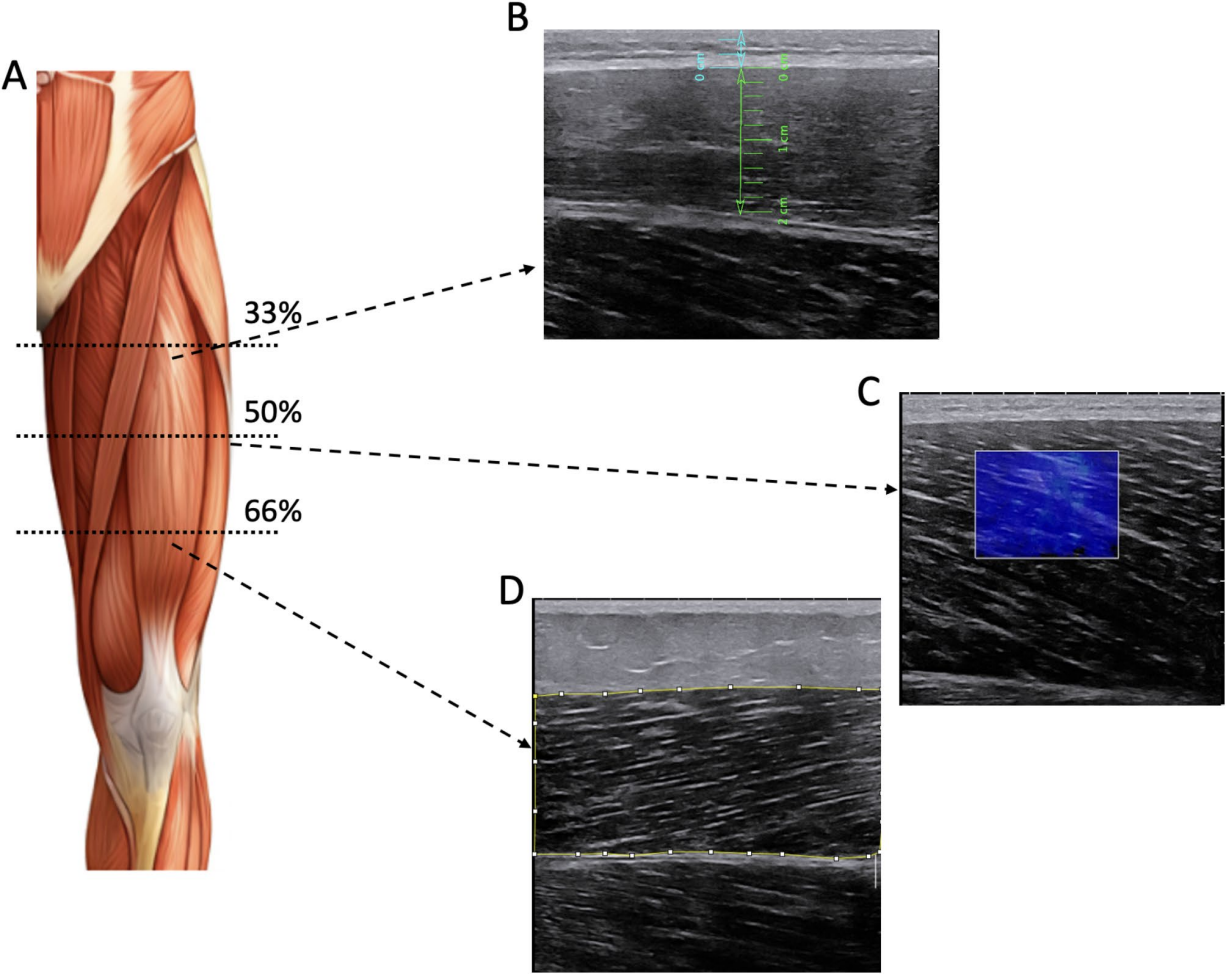
Examples of B-mode and shear-wave ultrasound images from different participants. (**A**) A schematic representation of the distances used to record images. (**B**) An example of a rectus femoris image recorded at the proximal region (33% of thigh length). The blue line represents the measurement of subcutaneous fat, and the green line indicates muscle thickness. (**C**) An example of a vastus lateralis image recorded at the mid-thigh (50% of thigh length) using shear wave mode to measure muscle stiffness. (**D**) An example of a rectus femoris image recorded at the distal region (66% of thigh length). The yellow line in the image indicates the region used to calculate gray-scale values for estimating muscle quality. The image of the leg (**A**) was generated using the DALL-E program.

 During 10 min, participants remained seated (knees at 90º of flexion, and hips at 100º) before ultrasound measurements. While they were seated, the anterior regions of the dominant thigh were marked to identify the reference points for the ultrasound image acquisition. Skin marks were made on the VL and RF muscles at 33% (distal), 50% (medial), and 66% (distal) of the distance from the anterior superior iliac spine to the lateral side of the patella^40^. Following skin marking, an evaluator (MBL) acquired all images. Two ultrasound images per site were obtained using B-mode ultrasonography (Aixplorer, Supersonic Imagine, Aix-en-Provence, France) with a SuperLinear 5–18 MHz frequency probe (5.5 cm). For all measurements, the probe was positioned longitudinally on both muscles (Fig. 3). Using the same ultrasound and probe, two additional images were acquired using shear-wave elastography mode (for muscle stiffness calculation) by using the General preset (SWE optimization: superficial, persistence: High, smoothing level: 5). An example of US images can be seen in Fig. 3.

#### Knee extensors MVIC

Participants were securely strapped at the trunk, hips, and leg to the isokinetic machine (Humac Norm, USA) to minimize extraneous bodily movement, and performed a warm-up. The knee was positioned at 90º of knee flexion, and hips at 100º. All tasks were isometric. The warm-up consisted of a series of submaximum contractions performed at 50% (×3), 75% (×2), and 90% (×1) of perceived maximum as reported somewhere^41^. For the MVIC, participants were instructed to perform a maximum effort as fast and as hard as possible for 5 s, with at least 30 s of rest between each attempt. To optimize participant performance, continuous biofeedback was supplied by positioning the dynamometer’s force production display in the participant’s view. Additionally, verbal motivation was consistently provided throughout each trial.

#### Evoke contractions

After completing the MVIC, participants were given a minimum rest period of 5 min before the evoked twitch responses were elicited^36^. This time course was dected as the point Transcutaneous femoral nerve stimulation was delivered with a constant current variable voltage stimulator (DS7AH, Digitimer Ltd., Welwyn Garden City, UK) while the participant remained passive^41^. After applying the conductive gel, a carbon rubber anode, sized 60 × 50 mm, was placed over the greater trochanter, and a carbon rubber cathode, measuring 35 × 25 mm, was aligned with the femoral nerve within the femoral triangle. A sequential repositioning of the cathode was carried out to ensure the site of the strongest twitch response; after this identification, the cathode was taped down. After that, incremental single-pulse stimuli with 200 µs square-wave pulses were delivered (every 15 s, in 20 mA increments) until the peak twitch force plateaued. Once the plateau was reached, two additional increments were administered to confirm that the plateau level had been achieved. Subsequently, three supra-maximal stimuli were delivered (with 15 s between each stimulus) at a current of 120% of the plateau level to measure the supra-maximal twitch force (TF).

### Data analysis

#### Ultrasound imaging

 For all ultrasound measurements detailed subsequently, the measurements were performed twice for each percentage distance on each image. This process was applied to both images taken, and the average value of the measurements was used for statistical analysis. This method was consistently employed for all percentage distances at which images were acquired for all the following variables. *Echo intensity (muscle quality)*: the ImageJ software, version 1.53a (National Institutes of Health, USA) was used for image analysis. Ultrasound images were exported from the ultrasound machine and imported to ImageJ in jpeg format. The region of interest of RF and VL was measured using the polygon tool by the same evaluator (SL) on both acquired images for all the distances. To maintain uniformity and precision in the analysis of echo intensity^21^, the images were displayed with identical settings for all participants, which included a gain of 62 dB, a depth of 6.0 cm, and a frequency of 15 Hz. The Intraclass Correlation (ICC_(2,1)_) for muscle quality between the images for each muscle and location were excellent, ranging from 0.88 to 0.92. *Muscle thickness (MT)*: the open-source Tracker software (version 6.13, physlets.org/tracker) was utilized for image analysis^39^. In summary, the ultrasound images were transferred in JPEG format from the imaging device to the dedicated software. Measurements of MT were consistently conducted from these images by the same evaluator (NF). For the RF and VL muscles, MT involved determining the distance between the upper and lower aponeuroses at each image. The ICC_(2,1)_ between the images for muscle thickness ranged from a minimum of 0.92 up to 0.97. *Muscle Stiffness*: after images were recorded, data analysis was performed using the ultrasound machine software by the same evaluator (RB). A circular region of interest of 10 mm in diameter was used to cover the muscles (VL and RF) and record the shear wave velocity (m/s)^42^. The ICC_(2,1)_ between the images for muscle stiffness ranged from minimum of 0.78 up to 0.92. For all variables presented here, a mean value of all two sites was used for further analysis.

#### Maximum voluntary isometric contraction (MVIC), rate of force development (RFD), and twitch force (TF)

Force signals from the isokinetic dynamometer were synced with Spike 2 software (CED, Cambridge, UK) for analysis. The force signal underwent low-pass filtering at a threshold of 500 Hz using a fourth-order zero-lag Butterworth digital filter, and to eliminate main frequency interference, a notch filter at 50 Hz with an infinite impulse response digital filter, featuring a quality factor of 10, was applied. Maximum force (MF) was calculated as the highest instantaneous point during the MVIC. Similarly, TF was calculated as the highest instantaneous point during the evoked contraction. RFD was measured from the onset of force to the peak force, and the value was then divided by the duration of the epoch. The onset of force was identified as previously recommended^43^.

### Statistical analysis

The statistical analyses were performed using SPSS version 29.0 (IBM Inc., Chicago, IL, USA). The level of significance was set at the α < 0.05, and data is presented as mean ± SD. It is important to highlight that echo intensity values have been reported to be influenced by subcutaneous fat^21^. Taking this into consideration, we tested the association between echo intensity and subcutaneous fat measurements in our sample, and there was no significant association between the two variables. Thus, no correction was made, and statistical analysis was performed using raw echo intensity. Data were first examined for normality (Shapiro-Wilk test) and homogeneity of variance (Levene’s test). For the study’s first aim, which was to analyze the differences between sites for muscle stiffness, muscle quality, MT, and SF, a one-way repeated measures ANOVA was used for each dependent variable. A Bonferroni-adjusted post-hoc test was employed when the main effect was detected in the ANOVA. Alternatively, non-normally distributed data were analyzed using the Friedman test and the Wilcoxon signed-rank test adjusted as the post hoc procedure. Cohen’s d effect sizes (d) were used to assess the magnitude of any effect and interpreted as follows: trivial, < 0.2; small, 0.2 to 0.49; medium, 0.50 to 0.79; and large, > 0.8^44^. For the study’s second aim, which was to explore the association between measures of force production (MF, RFD, and TF) and muscle morphology (MT, muscle stiffness, and muscle quality), Spearman’s rho correlation test was used.

## Data Availability

The datasets generated and/or analyzed in the current study are available upon reasonable request from the corresponding author.
